# Metabolic Profiling of an *Echinostoma caproni* Infection in the Mouse for Biomarker Discovery

**DOI:** 10.1371/journal.pntd.0000254

**Published:** 2008-07-02

**Authors:** Jasmina Saric, Jia V. Li, Yulan Wang, Jennifer Keiser, Jake G. Bundy, Elaine Holmes, Jürg Utzinger

**Affiliations:** 1 Department of Public Health and Epidemiology, Swiss Tropical Institute, Basel, Switzerland; 2 Department of Biomolecular Medicine, Division of Surgery, Oncology, Reproductive Biology and Anaesthetics (SORA), Faculty of Medicine, Imperial College London, London, United Kingdom; 3 Department of Medical Parasitology and Infection Biology, Swiss Tropical Institute, Basel, Switzerland; James Cook University, Australia

## Abstract

**Background:**

Metabolic profiling holds promise with regard to deepening our understanding of infection biology and disease states. The objectives of our study were to assess the global metabolic responses to an *Echinostoma caproni* infection in the mouse, and to compare the biomarkers extracted from different biofluids (plasma, stool, and urine) in terms of characterizing acute and chronic stages of this intestinal fluke infection.

**Methodology/Principal Findings:**

Twelve female NMRI mice were infected with 30 *E. caproni* metacercariae each. Plasma, stool, and urine samples were collected at 7 time points up to day 33 post-infection. Samples were also obtained from non-infected control mice at the same time points and measured using ^1^H nuclear magnetic resonance (NMR) spectroscopy. Spectral data were subjected to multivariate statistical analyses. In plasma and urine, an altered metabolic profile was already evident 1 day post-infection, characterized by reduced levels of plasma choline, acetate, formate, and lactate, coupled with increased levels of plasma glucose, and relatively lower concentrations of urinary creatine. The main changes in the urine metabolic profile started at day 8 post-infection, characterized by increased relative concentrations of trimethylamine and phenylacetylglycine and lower levels of 2-ketoisocaproate and showed differentiation over the course of the infection.

**Conclusion/Significance:**

The current investigation is part of a broader NMR-based metabonomics profiling strategy and confirms the utility of this approach for biomarker discovery. In the case of *E. caproni*, a diagnosis based on all three biofluids would deliver the most comprehensive fingerprint of an infection. For practical purposes, however, future diagnosis might aim at a single biofluid, in which case urine would be chosen for further investigation, based on quantity of biomarkers, ease of sampling, and the degree of differentiation from the non-infected control group.

## Author Summary

Consumption of raw fish and other freshwater products can lead to unpleasant worm infections. Indeed, such worm infections are of growing public health and veterinary concern, but they are often neglected, partially explained by the difficulty of accurate diagnosis. In the present study we infected 12 mice with an intestinal worm (i.e., *Echinostoma caproni*) and collected blood, stool, and urine samples 7 times between 1 and 33 days after the infection. At the same time points, blood, stool, and urine were also sampled from 12 uninfected mice. These biofluid samples were examined with a spectrometer and data were analyzed with a multivariate approach. We observed important differences between the infected and the uninfected control animals. For example, we found an increased level of branched chain amino acids in the stool of infected mice and subsequent depletion in blood plasma. Additionally, we observed changes related to a disturbed intestinal bacterial composition, particularly in urine and stool. The combination of results from the three types of biofluids gave the most comprehensive characterization of an *E. caproni* infection in the mouse. Urine would be the biofluid of choice for diagnosis of an infection because the ease of sample collection and the high number and extent of changed metabolites.

## Introduction

An estimated 40 million individuals are infected with food-borne trematodes and, in many parts of the world, the diseases caused by these infections are emerging [1]. Yet, food-borne trematodiases are so-called neglected tropical diseases [2]. An infection with food-borne trematodes is acquired by the consumption of the larval stage of the parasite, present in aquatic food products (e.g., freshwater fish, crustacean, and water plants). Adult flukes reside either in the intestine (e.g., *Echinostoma* spp.), the lung (e.g., *Paragonimus* spp.), or the liver (e.g., *Clonorchis sinensis*, *Fasciola* spp., *Opisthorchis* spp.) and can lead to various forms of pathology [2],[3].

A light infection with the intestinal fluke *Echinostoma* spp. in humans causes no marked deviation from the healthy state in the majority of cases, whereas the clinical symptoms due to a heavy infection include abdominal pain, violent diarrhea, anorexia, easy fatigue, and changes in the intestinal architecture, such as intestinal erosions, damage of intestinal mucosa, and catarrhal inflammation [4]. Histopathological investigations in mice and humans infected with *Echinostoma* spp. have revealed atrophied, fused and eroded villi, and a crypt hyperplasia in both lightly and heavily infected subjects [5]–[7].

At present, the most widely used diagnosis for infections with *Echinostoma* spp. and other food-borne trematodes, is by means of microscopic examination of stool samples for the presence of parasite eggs. However, light infection intensities, particularly at the onset of disease are often missed by this diagnostic approach. In addition, the detection of echinostome eggs in stool samples varies greatly due to species-dependent differences in egg laying capacity. Other means for diagnosis of food-borne trematode infections include immunological and molecular tests, such as the enzyme-linked immunosorbent assay (ELISA) [8] or polymerase chain reaction (PCR) [9], which depend on specificity of antigens and primers, respectively.

In the current study we applied a combination of ^1^H nuclear magnetic resonance (NMR) spectroscopy and multivariate statistical analysis to identify candidate biomarkers of an *E. caproni* infection and disease states in the mouse, by metabolic profiling of blood plasma, stool, and urine samples. *E. caproni* is a suitable trematode model that has been widely and effectively used in the laboratory for drug screening, and to deepen our understanding of the immunology and pathology of echinostomes and other food-borne trematodes in the vertebrate host [10]–[12]. NMR spectroscopy delivers a snapshot of the metabolite composition of biofluids, tissues and even bone, and has found a large array of applications in biology and medicine, such as the detection and differentiation of coronary heart disease [13], and biomarker identification in schizophrenia patients [14]. The systemic metabolic profile of a biological sample is of special interest, because it can be characteristic of the entire organism, and hence finds increasing application in systems biology [15]. The use of multivariate statistical methods to analyze and interpret complex spectral datasets makes it possible to deal with large sample data banks, and to detect differences between physiologically or pathologically distinct states. Candidate biomarkers can be identified from these models, taking into consideration intra-group variations, sample preparation methods, and spectral data acquisition. Thus far, we have characterized the global metabolic responses to several parasitic infections in rodents, namely (i) *Schistosoma mansoni* in the mouse [16], (ii) *Schistosoma japonicum* in the hamster [17], (iii) *Trichinella spiralis* in the mouse [18], (iv) *Trypanosoma brucei brucei* in the mouse [19], and (v) *Plasmodium berghei* in the mouse [56] mainly based on the urine and/or blood plasma metabolite profiles. Here we extend these initial host-parasite models to consider the relative merit of using biomarkers derived from a combined biological sample profile, and apply a metabolic profiling strategy for the first time to a food-borne trematode.

## Materials and Methods

### *E. caproni*-mouse model and animal husbandry

Our experiments were carried out in accordance with Swiss cantonal and national regulations on animal welfare (permission no. 2081). Female NMRI mice (n = 24) were purchased from RCC (Itingen, Switzerland), and housed in groups of 4 in macrolon cages under environmentally-controlled conditions (temperature: ∼25°C; humidity: ∼70%; light-dark cycle: 12–12 h). Mice had free access to commercially available rodent food from Nafag (Gossau, Switzerland) and community tap water supply.

Mice were 5 to 6-week-old at the onset of the experiments and had an average weight of 25.5 g (standard deviation (SD) = 0.9 g). Half of the mice remained uninfected throughout the study and served as controls. The other 12 mice were orally infected with 30 *E. caproni* metacercariae each (provided by B. Fried; Lafayette College, Easton, PA, United States of America) [20] on designated study day 0, which took place 1 week after arrival of animals to provide sufficient acclimatization time, and hence minimize stress-related impact on the metabolic profiles. Upon dissection of mice at the end of the experiment, however it was found that no infection had been established in 4 animals. Therefore these 4 mice were excluded from any further analysis.

### Collection of biofluids

Blood plasma, stool and urine samples were collected over a 33-day time course at 7 distinct sampling points (days 1, 5, 8, 12, 19, 26, and 33 post-infection), representative of different stages in the life of the *E. caproni* fluke, including acute and chronic infection stages. Collection was carried out between 08:00 and 10:00 hours in order to avoid potential variation of metabolite concentrations due to diurnal fluctuations. Stool and urine samples were collected into Petri dishes by gently rubbing the abdomen of the mice, and were immediately transferred into separate Eppendorf tubes and kept at −40°C. Blood samples (40–50 μl) were collected from the tail tip of each mouse into haematocrit tubes with sodium [Na] heparin-coat. Tubes were placed in a centrifuge (model 1–15, Sigma; Osterode am Harz, Germany) operated at 4,000 g for 4 min in order to separate plasma from red blood cells. The packed cell volume (PCV), i.e., length of red blood cells column in the microcapillary versus total length of blood sample column, was determined and expressed as percentage. Subsequently, the plasma fraction (∼20 μl) was transferred into a separate Eppendorf tube and kept at −40°C. Animals were weighed at each sampling point, using a Mettler balance (model K7T; Greifensee, Switzerland).

Mice were killed 36 days post-infection, using CO_2_. The small intestine was removed, and adult worms recovered from the ileum and jejunum and counted. Biological samples and an *E. caproni* specimen were forwarded to Imperial College London (United Kingdom) on dry ice and stored at −40°C prior to processing for ^1^H NMR spectroscopic data acquisition.

### Preparation of biofluids and *E. caproni* homogenate

Urine samples were prepared with a phosphate buffer (pH 7.4) containing 50% D_2_O (Goss Scientific Instruments; Chelmsford, United Kingdom) as a field frequency lock and 0.01% sodium 3-(trimethylsilyl) [2,2,3,3-^2^H_4_] propionate (TSP) (Cambridge Isotope Laboratories Inc.; Andover, MA, United States of America), as a chemical shift reference (δ 0.0). An aliquot of 25 μl of urine was added to 25 μl phosphate buffer. Plasma samples were prepared by adding 30 μl of 0.9% saline made up in 50% D_2_O into the Eppendorf tubes containing ∼20 μl of plasma. Because of the limited volumes of urine and plasma, samples were transferred into 1.7 mm diameter micro NMR-tubes (CortecNet; Paris, France) using a micro-syringe.

Stool samples were prepared with the same buffer as for urine but using 90% D_2_O to reduce the water content. Two pellets of stool were mashed with 700 μl buffer and sonicated for 30 min to inactivate gut bacteria and achieve biochemical stability in the sample. The samples were then centrifuged at 10,000 g for 2 min, and 550–600 μl of the supernatant was transferred into a new Eppendorf tube and stored at −40°C. Shortly before data acquisition, the stool supernatant was defrosted, centrifuged and transferred into NMR tubes of 5 mm outer diameter.

A tissue extraction was performed on the *E. caproni* specimen for ^1^H NMR spectroscopic analysis. The adult *E. caproni* fluke was mashed in 1 ml of chloroform with a glass mortar and pestle. A total of 1 ml of methanol and 1 ml of water were added, and this mixture was transferred into a glass tube. Another 0.5 ml of each liquid was used to rinse the mortar and transferred into the same glass tube. The mixture was centrifuged at 2,500 g for 30 min. The aqueous and the chloroform phases were transferred into a new glass tube each, chloroform was evaporated over night and the aqueous phase was lyophilized. Prior to ^1^H NMR data acquisition, the powder obtained from the aqueous phase was resolved in 550 μl phosphate buffer (90% D_2_O), whereas the dry mass of the chloroform fraction was dissolved in deuterated chloroform (CDCl_3_).

### Acquisition of spectral data

^1^H NMR spectra from plasma, stool, and urine samples, and the *E. caproni* extract were recorded on a Bruker DRX 600 NMR spectrometer, operating at 600.13 MHz for proton frequency (Bruker; Rheinstetten, Germany). A Bruker 5 mm triple resonance probe with inverse detection was used, employing a standard NMR 1-dimensional (1D) experiment with pulse sequence [recycle delay (RD)-90°-*t_1_*-90°-*t_m_*-90°-ACQ], setting *t_1_* to 3 μs, and using a mixing time (*t_m_*) of 150 ms. Water suppression was achieved with irradiation of the water peak during the RD set to 2 s and mixing time. The 90° pulse length was adjusted to ∼10 μs. A total of 256 transients were collected into ∼32,000 data points for each spectrum with a spectral width of 20 ppm. For plasma, two additional pulse programs were applied, namely Carr-Purcell-Meiboom-Gill (CPMG), and diffusion edited spectroscopy [21] to focus on the low and high molecular weight components of the plasma profile, respectively. All free induction decays (FIDs) were multiplied by an exponential function equivalent to a 0.3 Hz line-broadening factor prior to Fourier transformation.

Assignments of the spectral peaks were made from literature values [22]–[25] and confirmed *via* statistical total correlation spectroscopy (STOCSY) in MATLAB [26] and *via* standard 2-dimensional (2D) NMR experiments conducted on selected samples, including correlation spectroscopy (COSY), total correlation spectroscopy (TOCSY), and J-resolved NMR spectra [27],[28].

### Data processing and analysis

Data processing was as follows. First, spectra were corrected for phase and baseline distortions with an in-house developed MATLAB script. Second, the region containing the water/urea resonances (i.e., δ 4.2–6.3 in urine, δ 4.4–5.2 in plasma, and δ 4.7–5.5 in stool extracts) was excluded. Third, the spectra were normalized over the total sum of the remaining spectral area. Analysis of the spectral data was performed with principal component analysis (PCA) [29], projection to latent structure discriminant analysis (PLS-DA) and orthogonal (O)-PLS-DA [30]. PCA was used to explore any intrinsic similarity between samples. PCA models cannot be over-fitted since no prior information on infection status is included in the model. PLS-DA was then used to apply knowledge of infection status to optimize separation of classes and recovery of candidate biomarkers [30]. O-PLS-DA includes an orthogonal data filter in the PLS-DA and was used to further improve the extraction of infection-related biomarkers by removing the influence of systematic variation not related to infection status. The weight of contribution of the peaks is indicated by the color scale, whereby red symbolizes relatively high correlation with infection and blue indicates relatively low or no correlation. The metabolites which contributed the greatest weight to the O-PLS-DA coefficient plot were identified (Tables 1–3).

The NMR spectral data were used as the *X*-matrix and class information (infected or non-infected control) as the *Y*-matrix to build the O-PLS-DA models. A model consisting of one PLS component and one orthogonal component was generated using 7-fold cross validation.

Finally, in order to more accurately profile the temporal behavior of the discriminatory metabolites characterizing an *E. caproni* infection, computational integration was performed on selected resonances. Resonances from several of the metabolites in each sample, which showed infection-dependent variations, were integrated using an automated curve fitting program. The relative concentration in relation to the total spectral integral, subsequent to removal of the water resonance, was determined. This was performed in MATLAB using a previously published method [31], and further modified by a colleague (K. Veselkov; Imperial College London, UK). The p-values for the metabolites were assessed using a non-parametric 1-way analysis of variance (Mann-Whitney U) test in MATLAB.

## Results

### Physiological monitoring of mice

*E. caproni*-infected mice showed no visible sign of ill-health over the course of the experiment. The mean weight and mean PCV of *E. caproni*-infected (n = 8) and non-infected control mice (n = 12) did not differ at any of the time points investigated. The PCV values maintained a constant level throughout the experiment (49.6–55.1%). Upon dissection and worm count, an infection was confirmed in 8 out of the 12 mice (average worm count 26.5, SD = 12.0, range: 10–44 worms). The 4 animals with no established infection were excluded from further analyses.

### Composition of metabolite profiles

**Figure 1:**
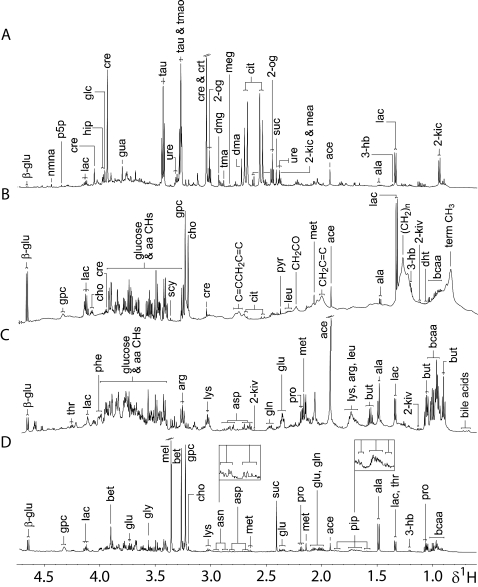
Aliphatic regions of representative 600 MHz ^1^H-NMR 1D spectra of biological samples obtained from an uninfected control mouse, aged 7–8 weeks. Spectra of urine (*A*), plasma (*B*) and fecal water (*C*) are shown. Additionally, the same region of a 600 MHz ^1^H NMR 1D spectrum of an *E. caproni* extract is depicted (*D*). Key: 2-og, 2-oxoglutarate; 3-hb, 3-hydroxybutyrate; 2-kic, 2-ketoisocaproate; 2-kiv, 2-ketoisovalerate; β-glu, β-glucose; aa, amino acids; ace, acetate; ala, alanine; arg, arginine; asn, asparagine; asp, aspartate; bcaa, branched chain amino acids; bet, betaine; but, butyrate; cit, citrate; cho, choline; cre, creatine; crt, creatinine; dht, dihydroxythymine; dma, dimethylamine; dmg, dimethylglycine; glc, glycolate; gln, glutamine; glu, glutamate; gly, glycine; gpc, glycerophosphocholine; gua, guanidinoacetate; hip, hippurate; lac, lactate; leu, leucine; lys, lysine; mea, methylamine; meg, methylguanidine; mel, methanol; met, methionine; nmna, *N*-methyl-nicotinamide; phe, phenylalanine; pip, pipecolate; pro, proline; p5p, pyridoxamine-5-phosphate; pyr, pyruvate; scy, *scyllo*-inositol; suc, succinate; tau, taurine; thr, threonine; tma, trimethylamine; tmao, trimethylamine-*N*-oxide; ure, ureidopropanoate.

**Figure 2:**
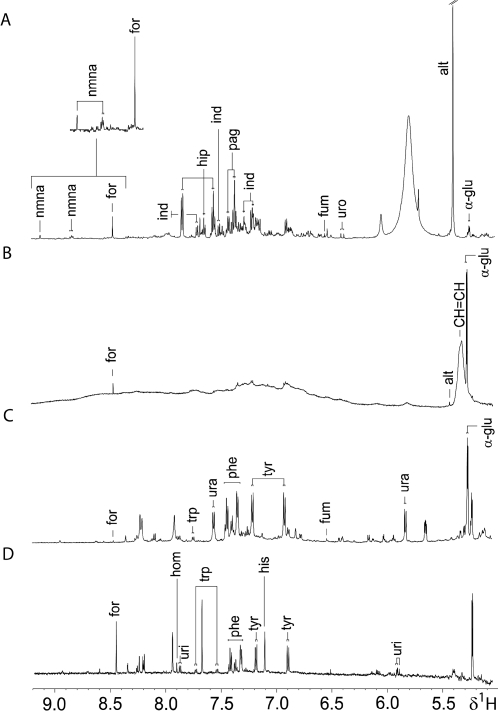
Aromatic regions of representative 600 MHz ^1^H-NMR 1D spectra of biological samples obtained from an uninfected control mouse, aged 7–8 weeks. Spectra of urine (*A*), plasma (*B*) and fecal water (*C*) are shown. Additionally, the same region of a 600 MHz ^1^H NMR 1D spectrum of an *E. caproni* extract is depicted (*D*). Key: α-glu, α-glucose; alt, alantoin; for, formate; fum, fumarate; hip, hippurate; his, histidine; hom, homocarnosine; ind, indoxylsulfate; nmna, *N*-methyl-nicotinamide; pag, phenylacetylglycine; phe, phenylalanine; trp, tryptophan; tyr, tyrosine; ura, uracil; uri, uridine; uro, urocanate.

Prior to assessing the metabolic effects of an *E. caproni* infection, the ^1^H NMR spectra of plasma, stool, and urine samples obtained from non-infected mice were characterized and found to be inherently different in composition. All three types of biofluids contained lactate, alanine, glucose, and acetate. Unique to the urine metabolic profile was the presence of hippurate, indoxylsulfate, urocanate, taurine, trimethylamine-*N*-oxide (TMAO), 2-oxoglutarate, ureidopropanoate, and 2-ketoisocaproate, amongst others (Figure 1A, Figure 2A and Table 1). The plasma spectral profiles were characterized by the predominance of various lipids and lipoprotein fractions, along with resonances from creatine, and several amino and organic acids (Figure 1B, Figure 2B and Table 2). Apart from the standard 1D acquisition, applied on all biofluids, a CPMG and diffusion edited pulse sequence was used in plasma profiling, to represent low and high molecular weight metabolites, respectively. Characteristic metabolic features of the stool extracts were bile acids and short chain fatty acids (SCFAs), such as butyrate, and propionate. In addition, other amino acids, such as tryptophan, lysine, arginine, and glutamine were more visible in stool spectra, compared to urine and plasma (Figure 1C, Figure 2C and Table 3).

**Table 1:** List of main metabolites found in urine over a 33-day study period in NMRI female mice.

Metabolite	Maximal time of metabolic change	Chemical moiety	Chemical shift in ppm and multiplicity
2-hydroxyisobutyrate		2×CH_3_	1.36(s)
2-ketoisocaproate	↓ (d12)	CH_2_, CH, 2×CH_3_	2.61(d), 2.10(m), 0.94(d)
2-oxoglutarate		β-CH_2_, γ-CH_2_	3.02(t), 2.50(t)
acetate	↓ (d8)	CH_3_	1.91(s)
alanine		α-CH, β-CH_3_	3.81(q), 1.48(d)
allantoin		CH	5.40(s)
citrate		1-CH_2_, 3-CH_2_	2.69(d), 2.54(d)
creatine	↓ (d8)	CH_3_, CH_2_	3.04(s), 3.93(s)
creatinine		CH_3_, CH_2_	3.05(s), 4.06(s)
dimethylamine		2×CH_3_	2.71(s)
dimethylglycine		2×CH_3_, CH_2_	2.89(s), 3.71(s)
formate		CH	8.45(s)
fumarate		CH	6.53(s)
α-glucose	↑ (d1); ↓ (d12)*	1-CH, 2-CH, 3-CH, 4-CH, 5-CH, half 6-CH_2_, half 6-CH_2_	5.24(d), 3.56(dd), 3.70(t), 3.40(t), 3.83(m), 3.72(dd), 3.85(m)
β-glucose	↑ (d1); ↓ (d12)*	1-CH, 2-CH, 3-CH, 4-CH, 5-CH, half 6-CH_2_, half 6-CH_2_	4.65(d), 3.25(dd), 3.47(t), 3.40(t), 3.47(ddd), 3.78(dd), 3.90(dd)
glycolate		CH_2_	3.94(s)
guanidinoacetate		CH_2_	3.80(s)
hippurate	↓ (d33)	CH_2_, 2,6-CH, 3,5-CH, 4-CH	3.97(d), 7.84(d), 7.55(t), 7.64(t)
indoxylsulfate		5-CH, 6-CH, 4-CH, 7-CH	7.20(t), 7.27(t), 7.51(d), 7.70(d)
lactate		CH, CH_3_	4.12(q), 1.33(d)
mannitol	↑ (d12)	2×α-CH_2_, 2×β-CH, 2×γ-CH	3.78(m), 3.88(dd), 3.68(dd)
methylcrotonate		β-CH, γ-CH_3_, γ′-CH_3_	1.66(s), 1.70(s), 1.71(s)
methylamine		CH_3_	2.61(s)
methylguanidine		CH_3_	2.83(s)
*N*-methyl-nicotinamide		CH_3_, 6-CH, 2-CH, 5-CH, 4-CH	4.48(s), 8.97(d), 9.28(s), 8.19(t), 8.90(d)
*p*-cresolglucuronide	↑ (d12)	2,6-CH, 3,5-CH, CH_3_	7.06(d), 7.23(d), 2.30(s)
phenylacetylglycine	↑ (d26)	2,6-CH, 3,5-CH, Ph-CH_2_, *N*-CH_2_	7.43(m), 7.37(m), 3.75(d), 3.68(s)
pyridoxamine-5-phosphate		OCH_2_, CH_2_N, CH_3_	7.67(s), 4.34(s), 2.48(s)
succinate	↑ (d33)	2×CH_2_	2.41(s)
taurine	↓ (d19)	CH_2_N, CH_2_S	3.27(t), 3.43(t)
trimethylamine	↑ (d12)	3×CH_3_	2.88(s)
trimethylamine-*N*-oxide	↑ (d12)	3×CH_3_	3.27(s)
ureidopropanoate		α-CH_2_, β-CH_2_	2.38(t), 3.3(t)
urocanate		α-CH, β-CH, 5-CH, 2-CH	6.40(d), 7.13(d), 7.41(s), 7.89(s)

The arrows show whether the metabolic change, associated with an *E. caproni* infection, is significantly increased (↑) or decreased (↓) in infected mice compared to non-infected control mice and the numbers, next to the arrows indicate the day of maximum significance. The p-values for the changing metabolites were assessed using a non-parametric 1-way analysis of variance (Mann-Whitney U) test in MATLAB, based on the integrals of the selected peaks and were all in the range of 0.001 to 0.05.

**Table 2 pntd-0000254-t002:** List of main plasma metabolites found in mice over a 33-day study period.

Metabolite	Maximal time of metabolic change	Chemical moiety	Chemical shift in ppm and multiplicity
2-ketoisovalerate		CH, 2×CH_3_	3.02(m), 1.13(d)
3-hydroxybutyrate		half α-CH_2_, half α-CH_2_, β-CH, γ-CH_3_	2.32(m), 2.42(m), 4.16(m), 1.21(d)
acetate	↑ (d12)	CH_3_	1.91(s)
acetoacetate		α-CH_2_, γ-CH_3_	2.29(s), 3.45(s)
alanine		α-CH, β-CH_3_	3.81(q), 1.48(d)
allantoin		CH	5.40(s)
choline	↓ (d33)	3×CH_3_, α-CH_2_, β-CH_2_	3.21(s), 4.07(m), 3.52(m)
citrate		1-CH_2_, 3-CH_2_	2.69(d), 2.54(d)
creatine	↓ (d12)	CH_3_, CH_2_	3.04(s), 3.93(s)
dihydroxythymine		CH_2_, CH, CH_3_	3.17(m), 2.47(m), 1.07(d)
formate	↑ (d12)	CH	8.45(s)
α-glucose	↑ (d1); ↓ (d12)*	1-CH, 2-CH, 3-CH, 4-CH, 5-CH, half 6-CH_2_, half 6-CH_2_	5.24(d), 3.56(dd), 3.70(t), 3.40(t), 3.83(m), 3.72(dd), 3.85(m)
β-glucose	↑ (d1); ↓ (d12)*	1-CH, 2-CH, 3-CH, 4-CH, 5-CH, half 6-CH_2_, half 6-CH_2_	4.65(d), 3.25(dd), 3.47(t), 3.40(t), 3.47(ddd), 3.78(dd), 3.90(dd)
glycerophosphocholine	↓ (d12)	3×CH_3_, half α-CH_2_, half α-CH_2_, half β-CH_2_, half β-CH_2_, γ-CH_2_	3.23(s), 4.32(t), 3.60(dd), 3.68(t), 3.89(m), 3.72(dd)
isoleucine	↓ (d33)	α-CH, β-CH, half γ-CH_2_, half γ-CH_2_, δ-CH_3_, β-CH_3_	3.68(d), 1.93(m), 1.25(m), 1.47(m), 0.99(d), 1.02(d)
lactate		CH, CH_3_	4.12(q), 1.33(d)
leucine	↓ (d33)	α-CH, β-CH_2_, γ-CH, δ-CH_3_, δ-CH_3_	3.72(t), 1.63(m), 1.69(m), 0.91(d), 0.94(d)
methionine		α-CH, β-CH_2_, γ-CH_2_, CH_3_	3.87(m), 2.10(m), 2.65(dd), 2.15(s)
*scyllo*-inositol		6×CH	3.35(s)
valine	↓ (d33)	α-CH, β-CH, γ-CH_3_, γ′-CH_3_	3.62(d), 2.28(m), 0.98(d), 1.03(d)
lipid fraction	↑	CH_3_	0.84(t)
lipid fraction	↑	(CH_2_)n	1.25(m)
lipid fraction	↑	β-CH_2_CH_2_CO	1.57(m)
lipid fraction	↑	CH_2_C = C	1.97(m), 2.00(m)
lipid fraction	↑	CH_2_CO	2.23(m)
lipid fraction	↑	C = CCH_2_C = C	2.69(m), 2.71(m), 2.72(m)
lipid fraction	↑	CH = CH	5.23(m), 5.26(m), 5.29(m)

Arrows indicate significantly changing substances comparing plasma of *E. caproni*-infected mice with non-infected control mice (↑, increased; ↓, decreased in infected animals) and the numbers in brackets indicate the day of maximum significance. ^*^glucose was the only metabolite found, which changed its directionality with time, i.e., it increased significantly after one day of infection and at day 12 post-infection was present in significantly lower concentrations, compared to uninfected control mice. The p-values for the changing metabolites were assessed using a non-parametric 1-way analysis of variance (Mann-Whitney U) test in MATLAB, based on the integrals of the selected peaks and were all in the range of 0.001 to 0.05.

**Table 3 pntd-0000254-t003:** Main metabolites found in fecal water of mice over a 33-day study period.

Metabolite	Maximal time of metabolic change	Chemical moiety	Chemical shift in ppm and multiplicity
2-hydroxyisovalerate		α-CH, β-CH, γ-CH_3_, γ′-CH_3_	3.85(d), 2.02(m), 0.79(d), 0.84(d)
2-ketoisocaproate		CH_2_, CH, 2×CH_3_	2.61(d), 2.10(m), 0.94(d)
2-ketoisovalerate		CH, 2×CH_3_	3.02(m), 1.13(d)
3-aminopropionic acid		α-CH_2_, β-CH_2_	2.56(t), 3.19(t)
3-hydroxyphenylpropionate		α-CH_2_, β-CH_2_, 2-CH	2.85(t), 2.47(m), 6.80(m)
2-oxoisoleucine		CH, half γ-CH_2_, half γ-CH_2_, δ-CH_3_, β-CH-CH_3_	2.93(m), 1.70(m), 1.46(m), 0.90(t), 1.10(d)
5-aminovalerate	↑ (d26)	5-CH_2_, 2-CH_2_, 3,4-CH_2_	3.02(t), 2.24(t), 1.65(m)
acetate	↓ (d12)	CH_3_	1.91(s)
alanine	↓ (d12)	α-CH, β-CH_3_	3.81(q), 1.48(d)
arginine		α-CH, β-CH_2_, γ-CH_2_, δ-CH_2_	3.76(t), 1.89(m), 1.59(m), 3.17(t)
asparagine		α-CH, half β-CH_2_, half β-CH_2_	4.01(m), 2.87(dd), 2.96(dd)
aspartate		α-CH, half β-CH_2_, half β-CH_2_	3.92(m), 2.70(m), 2.81(m)
bile acids		CH_3_	0.70(m)
butyrate	↓ (d26)	α-CH_2_, β-CH_2_, γ-CH_3_	2.16(t), 1.56(m), 0.90(t)
ethanolamine		NH-CH_2_, HO-CH_2_	3.15(t), 3.78(t)
formate		CH	8.45(s)
fumarate		CH	6.53(s)
α-glucose		1-CH, 2-CH, 3-CH, 4-CH, 5-CH, half 6-CH_2_, half 6-CH_2_	5.24(d), 3.56(dd), 3.70(t), 3.40(t), 3.83(m), 3.72(dd), 3.85(m)
β-glucose		1-CH, 2-CH, 3-CH, 4-CH, 5-CH, half 6-CH_2_, half 6-CH_2_	4.65(d), 3.25 (dd), 3.47(t), 3.40(t), 3.47(ddd), 3.78(dd), 3.90(dd)
glutamate		α-CH, β-CH_2_, γ-CH_2_	3.78(m), 2.06(m), 2.36(m)
glutamine		α-CH, β-CH_2_, γ-CH_2_	3.78(m), 2.15(m), 2.46(m)
glycerol		half α-CH_2_, half α-CH_2_, β-CH	3.56(dd), 3.64(dd), 3.87(m)
glycine	↓ (d12)	CH_2_	3.55(s)
hypoxanthine		3-CH, 7-CH	8.10(s), 8.11(s)
isoleucine	↑ (d26)	α-CH, β-CH, half γ-CH_2_, half γ-CH_2_, δ-CH_3_, β-CH_3_	3.68(d), 1.93(m), 1.25(m), 1.47(m), 0.99(d), 1.02(d)
lactate		CH, CH_3_	4.12(q), 1.33(d)
leucine	↑ (d8)	α-CH, β-CH_2_, γ-CH, δ-CH_3_, δ-CH_3_	3.72(t), 1.63(m), 1.69(m), 0.91(d), 0.94(d)
lysine		α-CH, β-CH_2_, γ-CH_2_, δ-CH_2_, ε-CH_2_	3.77(t), 1.92(m), 1.73(m), 1.47(m), 3.05(t)
methionine		α-CH, β-CH_2_, γ-CH_2_, CH_3_	3.87(m), 2.10(m), 2.65(dd), 2.15(s)
*myo*-inositol		1,3-CH, 2-CH, 5-CH, 4,6-CH	3.53(dd), 4.06(t), 3.28(t), 3.63(t)
phenylacetic acid		CH_2_, 2,4,6-CH, 3,5-CH	3.52(s), 7.29(t), 7.36(t)
phenylalanine		2,6-CH, 3,5-CH, 4-CH, half β-CH_2_, half β-CH_2_, α-CH	7.44(m), 7.39(m), 7.33(m), 3.17(dd), 3.30(dd), 3.99(dd)
proline		α-CH, half β-CH_2_, half β-CH_2_, γ-CH_2_, δ-CH_2_	4.15(dd), 2.05(m), 2.38(m), 2.00(m), 3.39(m)
propionate	↓ (d26)	CH_2_, CH_3_	2.19(q), 1.06(t)
succinate		2× CH_2_	2.41(s)
threonine		α-CH, β-CH, γ-CH_3_	3.60(d), 4.26(m), 1.33(d)
tryptophan		4-CH, 7-CH, 2-CH, 5-CH, 6-CH, α-CH, half β-CH_2_, half β-CH_2_	7.79(d), 7.56(d), 7.34(s), 7.29(t), 7.21(t), 4.06(dd), 3.49(dd), 3.31(dd)
tyrosine		2,6-CH, 3,5-CH, CH_2_, α-CH	7.23(d), 6.91(d), 2.93(t), 3.25(t)
uracil	↑ (d8)	5-CH, 6-CH	5.81(d), 7.59(d)
urocanate		α-CH, β-CH, 5-CH, 2-CH	6.40(d), 7.13(d), 7.41(s), 7.89(s)
valine	↑ (d26)	α-CH, β-CH, γ-CH_3_, γ′-CH_3_	3.62(d), 2.28(m), 0.98(d), 1.03(d)

Arrows indicate differences in the spectral profiles between *E. caproni*-infected mice and non-infected control mice (↑, increased; ↓, decreased in infected animals) and the numbers in brackets show the day post-infection of maximum concentration difference of the respective metabolite. The p-values for the changing metabolites were assessed using a non-parametric 1-way analysis of variance (Mann-Whitney U) test in MATLAB, based on the integrals of the selected peaks and were all in the range of 0.001 to 0.05.

In order to establish whether excretory products of the parasite itself were likely to contribute to any of the biofluids analyzed, a standard 1D spectrum of an adult *E. caproni* was acquired. The spectrum of the parasite differed from the biofluids obtained from the mouse host in content of homocarnosine, histidinol, uridine, pipecolate, and betaine (Figure 1D, Figure 2D), although betaine has been observed in ^1^H NMR spectra of rodent urine in previous studies [32]. Tables 1–4 summarize key metabolites found in urine, plasma, and stool extracts of mice, and in the *E. caproni* homogenate, respectively.

**Table 4:** List of main metabolites found in extracts of an adult *E. caproni*.

Metabolite	Chemical moiety	Chemical shift in ppm and multiplicity
3-hydroxybutyrate	half α-CH_2_, half α-CH_2_, β-CH, γ-CH_3_	2.32(m), 2.42(m), 4.16(m), 1.21(d)
acetate	CH_3_	1.91(s)
alanine	α-CH, β-CH_3_	3.81(q), 1.48(d)
betaine	CH_2_, CH_3_	3.90(s), 3.27(s)
choline	3×CH_3_, α-CH_2_, β-CH_2_	3.21(s), 4.07(m), 3.52(m)
formate	CH	8.45(s)
α-glucose	1-CH, 2-CH, 3-CH, 4-CH, 5-CH, half 6-CH_2_, half 6-CH_2_	5.24(d), 3.56(dd), 3.70(t), 3.40(t), 3.83(m), 3.72(dd), 3.85(m)
β-glucose	1-CH, 2-CH, 3-CH, 4-CH, 5-CH, half 6-CH_2_, half 6-CH_2_	4.65(d), 3.25(dd), 3.47(t), 3.40(t), 3.47(ddd), 3.78(dd), 3.90(dd)
glutamine	α-CH, β-CH_2_, γ-CH_2_	3.78(m), 2.15(m), 2.46(m)
glycerophosphocholine	3×CH_3_, half α-CH_2_, half α-CH_2_, half β-CH_2_, half β-CH_2_, γ-CH_2_	3.23(s), 4.32(t), 3.60(dd), 3.68(t), 3.89(m), 3.72(dd)
glycine	CH_2_	3.55(s)
histidinol	5-CH, 3-CH, γ-CH2, β-CH, α-CH	7.89(s), 7.12(s), 3.85(dd), 3.67(m), 3.62(m)
homocarnosine	5-CH, 3-CH, half ring-CH_2_, half ring-CH_2_, N-CH, N-CH_2_, CO-CH_2_, CH_2_	7.90(s), 7.01(s), 3.17(dd), 2.96(dd), 4.48(m), 2.92(m), 2.36(m), 1.89(m)
isoleucine	α-CH, β-CH, half γ-CH_2_, half γ-CH_2_, δ-CH_3_, β-CH_3_	3.68(d), 1.93(m), 1.25(m),1.47(m), 0.99(d), 1.02(d)
lactate	CH, CH_3_	4.12(q), 1.33(d)
leucine	α-CH, β-CH_2_, γ-CH, δ-CH_3_, δ-CH_3_	3.72(t), 1.63(m), 1.69(m), 0.91(d), 0.94(d)
lysine	α-CH, β-CH_2_, γ-CH_2_, δ-CH_2_, ε-CH_2_	3.77(t), 1.92(m), 1.73(m), 1.47(m), 3.05(t)
methionine	α-CH, β-CH_2_, γ-CH_2_, CH_3_	3.87(m), 2.10(m), 2.65(dd), 2.15(s)
phenylalanine	2,6-CH, 3,5-CH, 4-CH, half β-CH_2_, half β-CH_2_, α-CH	7.44(m), 7.39(m), 7.33(m), 3.17(dd), 3.30(dd), 3.99(dd)
pipecolate	half 3,4,5-CH_2_, half 4,5-CH_2_, half 3-CH_2_, half 6-CH_2_, half 6-CH_2_, 2-CH	1.60–1.66(m), 1.86(m), 2.22(m), 3.02(m), 3.43(m), 3.60(m)
proline	α-CH, half β-CH_2_, half β-CH_2_, γ-CH_2_, δ-CH_2_	4.15(dd), 2.05(m), 2.38(m), 2.00(m), 3.39(m)
propionate	CH_2_, CH_3_	2.19(q), 1.06(t)
*scyllo*-inositol	6×CH	3.35(s)
succinate	2×CH_2_	2.41(s)
threonine	α-CH, β-CH, γ-CH_3_	3.60(d), 4.26(m), 1.33(d)
tryptophan	4-CH, 7-CH, 2-CH, 5-CH, 6-CH, α-CH, half β-CH_2_, half β-CH_2_	7.79(d), 7.56(d), 7.34(s), 7.29(t), 7.21(t), 4.06(dd), 3.49(dd), 3.31(dd)
tyrosine	2,6-CH, 3,5-CH, CH_2_, α-CH	7.23(d), 6.91(d), 2.93(t), 3.25(t)
uridine	6-CH, 5-CH, 2′-CH, 3′-CH, 4′-CH, 5′-CH(d), half CH_2_OH, half CH_2_OH	7.87(d), 5.90(s), 5.92(d), 4.36(t), 4.24(t), 4.14(q), 3.92(dd), 3.81(dd)
valine	α-CH, β-CH, γ-CH_3_, γ′-CH_3_	3.62(d), 2.28(m), 0.98(d), 1.03(d)

### Multivariate analysis and global metabolic trajectories

**Figure 3:**
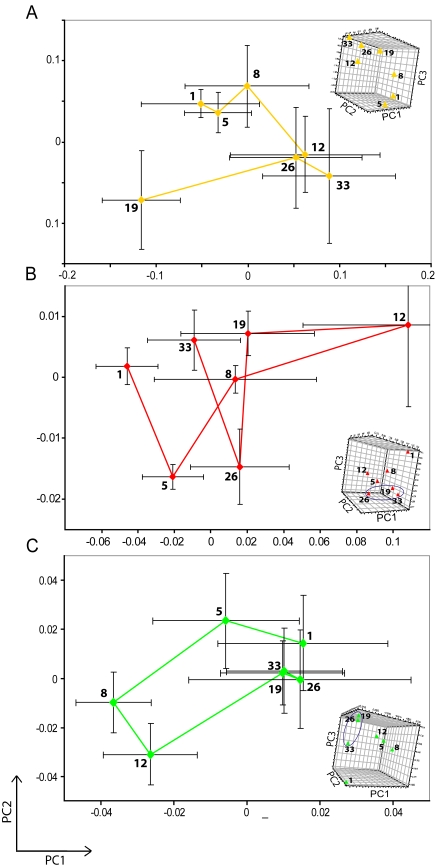
PCA trajectory plot of urine (*A*), plasma (*B*) and fecal water spectra (*C*) obtained from the mean PC1 and PC2 values for the *E. caproni*-infected mice over a 33-day period. The collection of the biofluids was performed at days 1, 5, 8, 12, 19, 26, and 33 post-infection. The ellipses in the 3D plots (Figures 3B and C) are for illustration purposes only to denote time points which become distinct from controls in 3D but are not clearly resolved in two dimensions, and are not based on statistical boundaries.

In both PCA and PLS-DA scores plots of the urinary metabolite profiles, a clear separation of *E. caproni*-infected and non-infected control mice was already visible 1 day post-infection. This separation was maintained in all later time points except day 5. Metabolic trajectories were constructed for each type of biofluid by taking the mean position in the principal component (PC) scores plot for each group of mice (*E. caproni*-infected and non-infected controls) separately, and connecting the coordinates chronologically to establish any systematic change in metabolic composition over the time course of the experiment. The control group showed no significant movement over the study duration (data not shown), whereas in the infected group, day 19 was significantly separated from all other days post-infection, and the whole time course of infection showed a shift from the upper left to the lower right quadrant (Figure 3A), whereby days 1 and 5 post-infection differed significantly from the sampling end point (day 33).

The plasma spectra of *E. caproni*-infected mice showed marked differences at days 1, 12, 26, and 33 post-infection, with the best discriminatory model at day 12 post-infection (goodness of prediction (Q^2^) according to PCA = 0.97; Q^2^Y (PLS-DA) = 0.89). Comparing the 3 different pulse programs applied, plasma time trajectories, showed similar behavior. The control trajectories were generally clustered, indicating stability of the metabolite composition over the study period. However, the standard 1D trajectory showed a slight difference between early and late time points (e.g., day 1 was separated in space from days 19 to 33). In contrast, for the *E. caproni*-infected animals, there was a significant metabolic movement from early (day 1) to intermediate time points post-infection (days 5 and 12) in the first component and finally to late time points (days 26 and 33) in the third component (Figure 3B). This movement pattern was consistent across the datasets acquired by all three pulse programs.

With regard to the ^1^H NMR spectra obtained from stool samples, a clear separation was found at day 5 post-infection in both the PCA and PLS-DA scores plot between *E. caproni*-infected and non-infected control mice, with maximum model fit for the PLS-DA model at day 26 post-infection (Q^2^ = 0.79). At the final time point (day 33), the two groups were metabolically similar; there was no separation between infected and non-infected mice using PCA, and the PLS-DA model revealed a lower, but still significant Q^2^ value than all previous time points. By comparing the time trajectories of the non-infected control with the *E. caproni*-infected group of mice, the controls were more tightly clustered, but showed a significant movement from day 8 post-infection onwards along PC1. In the 2D time trajectory plot of the *E. caproni*-infected mice, all time points were tightly clustered with the exception of days 8 and 12 post-infection, which comprised a separate cluster (Figure 3C). Introducing an additional PC brought about clear differentiation of the late time points (days 19, 26, and 33) from day 1 post-infection caused by more subtle systematic variation in metabolic levels.

### Pair wise comparison of time points across different types of biofluids

**Figure 4:**
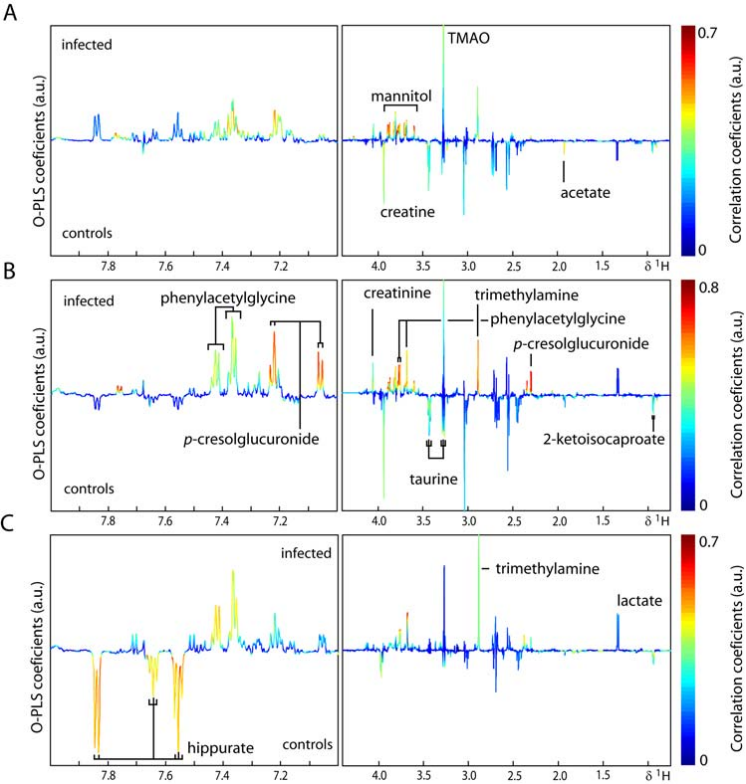
Pair-wise comparison *via* O-PLS-DA between urine obtained from non-infected control mice (control) and *E. caproni*-infected mice (infected) at 3 different sampling time points, i.e., day 8 (*A*), day 12 (*B*) and day 33 (*C*) post-infection. The color scale indicates the relative contribution of the peak/region to the strength of the differentiation model and the peak intensity is measured relative to the whole peak contribution in arbitrary units (a.u.). Note that the aromatic region (left part) is magnified by a factor 5.

O-PLS-DA was used to extract information on specific metabolic changes induced by an *E. caproni* infection over the duration of the study. Changes in urinary, plasma and fecal metabolites are presented in Figures 4–6 for selected time points and the complete set are summarized in Figure 7. Amongst the most significantly changed urinary metabolites were hippurate (decreased at day 33), 2-ketoisocaproate (decreased from day 8 onwards), trimethylamine (TMA; increased at days 8, 12, 19, and 33), taurine (decreased at days 8, 12, and 19), *p*-cresol glucuronide (increased at days 8, 12, 19, and 26), mannitol (increased from day 5 onwards), TMAO (increased at days 8, and 12), phenylacetylglycine (increased from day 12 onwards), acetate (decreased at days 8, and 19) and creatine (decreased at days 1, 5, 8, and 12).

**Figure 5 pntd-0000254-g005:**
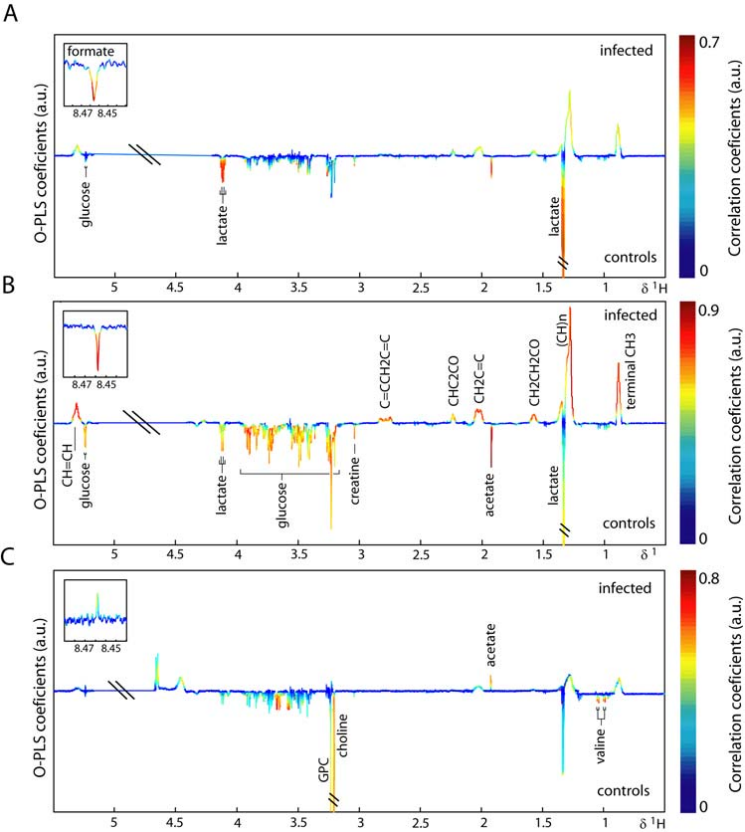
Pair-wise comparison *via* O-PLS-DA between plasma CPMG spectra obtained from non-infected control mice (control) and *E. caproni*-infected mice (infected) at 3 different sampling time points, i.e., day 8 (*A*), day 12 (*B*) and day 33 (*C*) post-infection. The color scale indicates the relative contribution of the peak/region to the strength of the differentiation model and the peak intensity is measured relative to the whole peak contribution in arbitrary units (a.u.). The CPMG spectrum represents small molecular weight components.

**Figure 6:**
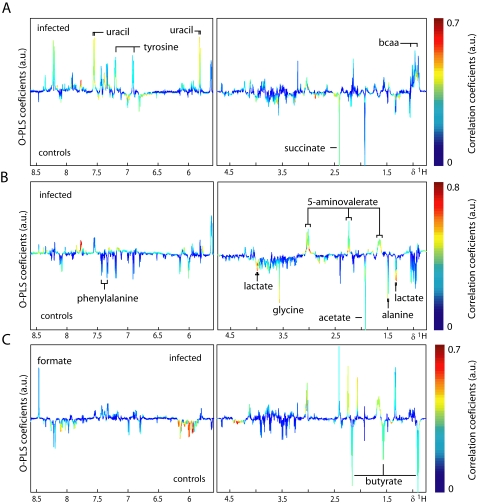
Pair-wise comparison *via* O-PLS-DA between fecal water spectra obtained from non-infected control mice (control) and *E. caproni*-infected mice (infected) at 3 different sampling time points, i.e., day 8 (*A*), day 12 (*B*) and day 33 (*C*) post-infection. The color scale indicates the relative contribution of the peak/region to the strength of the differentiation model and the peak intensity is measured relative to the whole peak contribution in arbitrary units (a.u.). Note that the aromatic region (left part) is magnified by a factor 5.

Plasma from infected mice showed changes in the relative concentration of acetate (increased at all time points except day 5), creatine (decreased from day 8 onwards), lipids (increased from day 8 onwards), formate (decreased at days 1, 8, 12, and 19, but increased at day 33), lactate (decreased at days 1, 8, 12, 19, and 26), glucose (increased at days 1, and 33, but decreased at days 12, 19, and 26), glycerophosphorylcholine (GPC; decreased at days 12, 26, and 33), choline (decreased at days 1, 12, 26, and 33) and branched chain amino acids (BCAAs; decreased at days 12, 26, and 33).

The changes in stool samples from infected animals included the BCAAs (increased at days 8, and 26), uracil (increased at day 8), butyrate (decreased at days 12, 19, and 26), propionate (decreased at days 12, 19, and 26) and 5-aminovalerate (increased from day 5 onwards).

### Analysis of relative concentrations of key metabolites over study duration

Figure 8 shows the relative concentration of some of these metabolites both for control (blue) and infected mice (colored according to biofluid). The error bars signify 2 SDs of the mean. According to this 3×3 diagram, the selected plasma and urine metabolites showed a more robust pattern of group separation over time when compared to fecal water extracts. Whereas some overlap was observed in the scores plot relating to the fecal water samples, there was a tendency toward increasing discrimination of urinary metabolites with time over the course of an *E. caproni* infection, whereas the discrimination became smaller toward the end of the experiment in the selected plasma metabolites.

## Discussion

^1^H NMR-based metabolic profiling of biofluids is an established method for deepening our understanding of host-parasite interactions and for investigating disease states in clinical studies [15],[19],[33]. Sample preparation and spectral acquisition of a biofluid takes little time, and often an overview of the metabolic state of the organism can be obtained by visual assessment of the spectra. Identification of biomarkers from different types of biofluids is similarly convenient, although stool samples need slightly more preparation time and require sonication and an additional centrifugation step, due to the high amount of sediments in the stool-buffer mixture [34].

**Figure 7:**
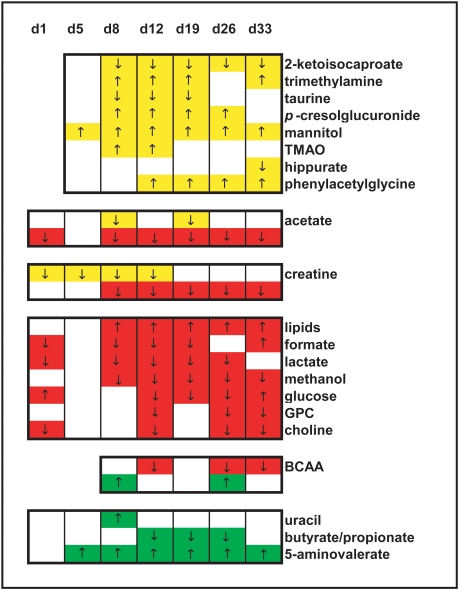
Summary of key *E. caproni* infection-related features for urine (yellow), plasma (red), and fecal water (green). The colored regions show the differences between *E. caproni*-infected mice and non-infected control animals, whereas the changes in direction are indicated by arrows (↑, indicates an increase in the metabolite signal in infected mice with respect to the control group; ↓, indicates a decreased metabolite signal in infected mice).

**Figure 8:**
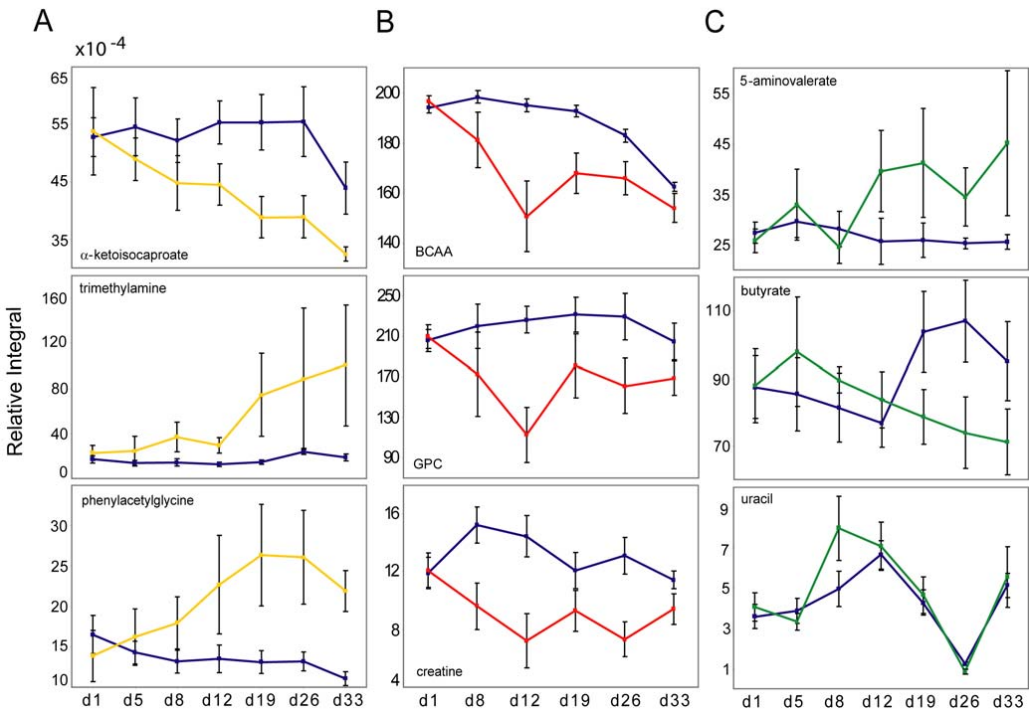
Statistical integration for 3 selected metabolites in the spectra of each of the 3 different types of biofluids (*A*, urine; *B*, plasma; *C*, fecal water). The relative concentration with respect to the total spectral area of each of these metabolites is shown for non-infected control mice (blue), and *E. caproni*-infected mice (yellow representing urine, red representing plasma, and green representing fecal water). The error bars signify 2 standard deviations of the mean.

A number of potential biomarkers for diagnosis of an *E. caproni* infection in the mouse were found here for each of the biofluids employed; 12 in plasma, 10 in urine and 7 in stool. Hence, if for practical purpose, a diagnosis was required based on a single biofluid, either plasma or urine would be the first choice for further development. Stool is the least suitable biological sample not only in terms of a lower number of potential biomarkers, but also because of the more difficult sample preparation, a lower robustness of metabolites (Figures 7 and 8), and larger inter- and intra-individual variation [34]. The latter issue makes it difficult to determine if the change is related to the actual infection, or results from other factors, e.g., age and/or microbiotal presence or activity. Although the high degree of individual variation in the fecal metabolite profiles derived from laboratory studies can be overcome with parallel monitoring of metabolic time-related changes in a control group, this issue needs to be addressed in future applications of metabonomics for diagnosis of parasitic infections and disease states, and hence for monitoring disease control programs. If the stability of the urine profiles compared to plasma over the study duration is taken into account, then urine would be the biofluid of choice on which to base a diagnostic. Morevoer, urine collection is less invasive than blood collection.

### Inter-group separation and trajectories

The time trajectories allow the influence of growth and maturation of either host or parasite to be considered. Since very few of the metabolites observed in the *E. caproni* fluke appeared in any biofluid, infection-related changes are unlikely to correlate with maturation of the parasite. Additionally, since the urinary time trajectories were very different, comparing the control with the infected group, whereas the control trajectory did not show any significant movement over time; this would indicate that the maturation of the host organism did not markedly influence the metabolic profile over the 33-day time course. Hence, the systemic movement over time observed in the infected group is most likely to be related to the establishment and progression of the *E. caproni* infection.

In stool and plasma, the time trajectories of the infected animals demonstrated a markedly greater magnitude from the baseline position than the non-infected animals. The greatest differentiation between *E. caproni*-infected mice and non-infected control animals was found in the PC scores plots based on the urine spectral profiles. The smallest differentiation was observed in the stool. From the scale of the PC scores plot axes the trajectory of the infected group occupied a 1.5 and 50 times larger space for stool and plasma, respectively than the non-infected group, whereas in urine, the control trajectory occupied a 10^6^ times bigger space, compared to the trajectory of infection. The magnitude of infection-induced metabolic disturbance in the urine profile again clearly points to the greater suitability of urine as a diagnostic biological matrix.

### Intestinal re-absorption

The considerable increase in concentration of lipids in the plasma, e.g., fatty acids, triaglycerols, and lipoproteins, reflects the action of the parasite in the hosts gut. In mice harboring a 2-week-old *E. caproni*-infection, an increased breakdown of membrane lipids in the host intestinal tissue has been observed [35], which is consistent with the present findings of a maximum lipid increase on day 12 post-infection [36]. The excretory products of *E. caproni* in the intestinal mucosa are primarily free sterols, triaglycerols, and free fatty acids [37], but it is unlikely that the amounts excreted by the parasite make a substantial contribution to the host metabolic profile, given that the total parasite mass to host weight ration is ∼1:300.

Whilst the simple diffusion of lipid micelles into mucosal cells seems unaffected by the parasite, the Na^+^-dependent active transport of amino acids could be impaired as the increase of the BCAAs in stool as the subsequent decrease in plasma supports. Depletion of the carrier molecules at the brush border of the mucosal cells, or a change of the electrochemical gradient for Na^+^ might explain the selective impact on trans-luminal gut transport [38].

The observed decrease of leucine in plasma, in turn, might induce the significant reduction in levels of 2-ketoisocaproate in urine, which is a transamination product of the former [39]. Taurine is mainly conjugated with cholic acid and chenodeoxycholic acid in the liver to form primary bile salts, and is excreted *via* the urine after deconjugation from the bile salt or it leads into the sulphur- or pyruvate metabolism. Once the taurine conjugated bile salt has transformed the lipids into a micellar form, which is necessary to cross the intestinal wall, taurine is deconjugated by gut bacterial species and reabsorbed into the liver *via* blood circulation [40]. The decreased levels of excreted taurine in the urine of the infected mice may result from the higher demand for increased lipid digestion, resulting from the action of *E. caproni* in the gut.

### Gut microbiota

The changes in hippurate, phenylacetylglycine, *p*-cresol-glucuronide, and TMA in urine, and 5-aminovalerate, and the SCFA levels in stool, are associated with a change in gut microbiotal presence or activity, as all of these metabolites undergo modification *via* gut microbial species before excretion. For instance, 5-aminovalerate is formed by several different *Clostridium* species which utilize ornithine and proline as substrates, but to our knowledge, only *C. aminovalericum* degrades 5-aminovalerate further to form mainly propionate and acetate [41]–[43]. This may imply that the presence of *E. caproni* in the gut disturbs the microbial balance resulting in depleted or inactivated *C. aminovalericum.* The formation of *p*-cresol is likewise known to be performed by a *Clostridium* subspecies (i.e., *C. difficile* and *C. scatologenes*) [44],[45], with the bacterium-specific enzyme *p*-hydroxyphenylacetate. It is then conceivable that *p*-cresol is taken up by the bloodstream, bound to serum proteins and glucuronidated in the kidney prior to excretion [46]. Increased excretion of *p*-cresol-glucuronide might be coupled with a higher activity or higher presence of this bacterial strain as the kidney function does not seem to be impaired in infected animals. PCR analyses on several different *Clostridium* sub-strains are ongoing and will be discussed in forthcoming publications.

The decrease of the SCFAs in stool may also be indicative of an unbalanced microbiota, as dietary carbohydrates (e.g., starches and fibres) are fermented by colonic bacteria to mainly acetate, propionate, and butyrate. Whilst butyrate serves as main energy source for colonocytes, acetate and propionate pass through the intestinal wall and move *via* peripheral blood to the liver where they have antagonistic functions on the cholesterol synthesis. Whilst the former increases cholesterol synthesis, the latter was shown to act as an inhibitor. The uptake from colon by the blood system is four times higher in the case of acetate, than propionate, which is a possible explanation for depletion of the SCFA, also reflected by the observed decrease in levels of acetate in both urine and plasma [47],[48].

The increased concentration of TMA and phenylacetylglycine, and the decrease of hippurate in urine, observed at the later time points of our experiment, are concomitant phenomena of the changed gut microbiota [49],[50]. Trimethylammonium compounds like choline and carnitine, which are ingested in the normal diet, are degraded by intestinal bacteria to TMA, and then oxidized in the liver to TMAO in a second step [51],[52]. A microbial shift toward choline degrading bacteria might explain the choline/GPC depletion in plasma and the subsequent increase of TMAO in urine.

### Specificity of biomarkers for diagnosis

An infection with *E. caproni* induces changes in the concentration of a range of metabolites in urine, plasma, and stool. To be useful as a ‘real’ biomarker, the metabolic candidate must be reproducible, robust, specific and, ideally, easy to measure [53]. From the current analyses an anomalous increase in urinary mannitol was noted in infected animals. Mannitol is likely to derive from the diet, since it is not synthesized by vertebrates. However, the higher amounts of urinary mannitol in *E. caproni*-infected mice, may reflect the higher intestinal permeability, compared to the control group [54].

To assess the specificity of the biomarkers identified for potential diagnosis of infection, the obtained *E. caproni* fingerprint was compared to altered metabolite patterns, associated with other parasite-rodent models [16]–[18]. Interestingly, *E. caproni* seems to alter the gut microbiota in a similar way to the biologically-related blood flukes, i.e., *S. mansoni* [16], and *S. japonicum* [17]. Hippurate, phenylacetylglycine, and TMA are modified by several gut microbial species before excretion in urine. In the 3 disease models, hippurate was found to decrease significantly, whereas phenylacetylglycine, *p*-cresol-glucuronide and TMA showed increased levels in infections with all 3 parasites, which suggests a common trematode-inherent influence on gut microbial composition. The change in 2-ketoisocaproate in urine was unique to the infection with *E. caproni* and 5-aminovalerate in stool may also deliver an *E. caproni*-specific marker but, at the time being, cannot be compared to other disease models, as the metabonomic assessment of stool was applied only in the present parasite-rodent model. The metabolic effect of a nematode infection (*T. spiralis*) in NIH Swiss mice has also been reported by Martin and colleagues [18]. *T. spiralis* has a similar initial mechanism of pathogenicity and also induces a state of inflammation of the gut before it migrates from the intestine to muscle tissue and induces hypercontractility [55]. While comparison between the study conducted by Martin *et al.* [18] and the current study revealed a number of biomarkers, which were the same in both models, the directionality of these metabolites was different. For example, a decrease in choline and creatine concentrations was observed in *E. caproni*-infected mice, whereas the same metabolites were reported to be increased in *T. spiralis*-infected mice compared to non-infected controls. Furthermore, the lipids (e.g., triaglycerides, saturated and unsaturated fatty acids) which undergo a marked increase in *E. caproni*-infected animals, showed a significant decrease in the mice infected with *T. spiralis*.

Future studies evaluating additional laboratory host-parasite models, and applying complementary metabolic profiling methods, such as ultra performance liquid chromatography (UPLC), in combination with mass spectrometry (MS), will help to confirm the specificity of the metabolic perturbations associated with an *E. caproni* infection. In conclusion, we have shown that metabolic profiling of plasma, urine, and stool delivers a comprehensive fingerprint of an *E. caproni* infection, composed of general as well as highly specific biomarkers (e.g., 2-ketoisocaproate and 5-aminovalerate). Keeping in mind the long-term objective of developing novel diagnostic assays for trematode-borne diseases, one would emphasize the value of further development, particularly based on the urine profiles.

## Supporting Information

Translation of the Abstract into German by Jasmina Saric (0.02 MB DOC)

Translation of the Abstract into Chinese by Yulan Wang (0.16 MB PDF)
