# Supplementary material for: Metabolic Profiling of an Echinostoma caproni Infection in the Mouse for Biomarker Discovery
Source: PLoS Negl Trop Dis. 2008 Jul 2;2(7):e254. doi: 10.1371/journal.pntd.0000254 (PMC2432044; doi:10.1371/journal.pntd.0000254)
Supplement: Translation of the Abstract into German by Jasmina Saric — (0.02 MB DOC) [file pntd.0000254.s001.doc]

*Translation of the abstract into German by Jasmina Saric (08-PNTD-RA-0042)*

**Charakterisierung einer *Echinostoma caproni* Infektion in der Maus mittels metabolischer Profile für die Entdeckung von Biomarkern**

**Zusammenfassung**

***Hintergrund:*** Metabolische Profile beinhalten wichtige Informationen, die uns erlauben biochemische Prozesse von Infektionskrankheiten und anderen pathologischen Zuständen besser zu verstehen. Das Ziel dieser Studie war es, die umfassende metabolische Reaktion auf eine Infektion mit dem intestinalen Trematoden *Echinostoma caproni* in der Maus zu untersuchen und die Biomarker, extrahiert aus Blut Plasma, Urin und Stuhl, zu vergleichen und auf ihr diagnostisches Potential in der akuten und chronischen Phase der Infektion zu evaluieren.

***Methoden/Hauptergebnisse:*** Zwölf weibliche NMRI Mäuse wurden mit jeweils 30 *E. caproni* Metazerkarien infiziert. Aus den Mäusen wurde Blut Plasma, Stuhl und Urine entnommen und zwar an 7 verschiedenen Zeitpunkten nach der Infektion. Gleichzeitig wurden Proben von einer nicht-infizierten Kontrollgruppe gesammelt. Alle Daten wurden mittels Proton Kernmagnetischer Resonanzspektroskopie (^1^H NMR) akquiriert und mit verschiedenen multivariaten statistischen Methoden analysiert. Die metabolischen Profile im Plasma und Urin zeigten schon einen Tag nach Infektion Veränderungen auf, wie zum Beispiel reduzierte Choline, Acetat, Format und Laktat Spiegel und erhöhte Glukose Spiegel im Plasma und tiefere Konzentrationen von Harn-Kreatin.

***Schlussfolgerung/Bedeutung:*** Diese Arbeit ist Teil einer umfassenden Studie die prüfen soll, ob sich die angewandten Methoden eignen, um krankheitsspezifische Biomarker im Verlaufe einer Parasiteninfektion aufzuspüren. Im Falle von *E. caproni* wäre eine Diagnose, basierend auf Untersuchungen von Plasma, Urin und Stuhl die Umfassendste und würde die Infektion am besten charakterisieren. Praktischerweise würde man sich jedoch auf ein einzelnes Untersuchungsmaterial festlegen, wobei Urin, durch die Quantität an gefundenen Biomarkern, die Einfachheit der Kollektion und den allgemeinen Grad an Differenzierung von der Kontrollgruppe, am besten geeignet wäre.
