# Supplementary material for: Metabolic Profiling of an Echinostoma caproni Infection in the Mouse for Biomarker Discovery
Source: PLoS Negl Trop Dis. 2008 Jul 2;2(7):e254. doi: 10.1371/journal.pntd.0000254 (PMC2432044; doi:10.1371/journal.pntd.0000254)
Supplement: Translation of the Abstract into Chinese by Yulan Wang — (0.16 MB PDF) [file pntd.0000254.s002.pdf]

摘要

背景：代谢指纹图谱可以帮助我们进一步理解传染病的发病机理。本项目的研究目的是在系统的水平上了解棘口属的 *E caproni* 的吸虫（*E. caproni*）对小鼠代谢的影响；同时通过比较不同生物样品（血浆、粪样和尿样）的代谢标记物，找出最适合描述急性和慢性感染的生物样品。

方法/结果：24 只小鼠(NMRI种系，雌，5-6 周），其中 12 只各感染 30 只*E. caproni* 尾蚴。分别从感染小鼠及 12 只对照小鼠中采集血浆、粪样和尿样，共采集了 7 个时间点（感染后第 1, 5, 8, 12, 19, 26 和 33 天)，经处理后获取^{1}H核磁共振图谱数据。通过多变量指纹图谱的数据分析发现：小鼠血浆和尿样中的代谢产物在感染后的第一天就发生了变化，主要是由于血浆中胆碱、醋酸、蚁酸和乳酸的降低及葡萄糖的增加而引起的。同时发现尿样中的肌酸降低了。

结论：验证代谢组学方法是寻找代谢标记物的有效方法是本项目的目的之一。通过分析三种生物样品来确定代谢标记物是比较全面的，但是从应用的角度考虑一种是比较适合的。从研究结果中发现：尿样是可以提供较稳定、更能识别感染代谢标记物的生物样品。

关键词：棘口属的 *E caproni* 的吸虫，诊断，核磁共振，代谢组学，代谢标记物，多变量指纹图谱的数据分析
